# Investigating rapid diagnostic testing in Kenya’s health system, 2018–2020: validating non-reporting in routine data using a health facility service assessment survey

**DOI:** 10.1186/s12913-023-09296-9

**Published:** 2023-03-30

**Authors:** Angela K Moturi, Bibian N Robert, Felix Bahati, Peter M Macharia, Emelda A Okiro

**Affiliations:** 1 Population Health Unit, Kenya Medical Research Institute-Wellcome Trust Research Programme, Nairobi, Kenya; 2 Health Services Research Unit, Kenya Medical Research Institute-Wellcome Trust Research Programme, Nairobi, Kenya; 3 Centre for Health Informatics, Computing, and Statistics, Lancaster Medical School, Lancaster University, Lancaster, UK; 4 Centre for Tropical Medicine and Global Health, Nuffield Department of Medicine, University of Oxford, Oxford, UK

**Keywords:** Rapid diagnostic test, Routine data, Health facility survey, Triangulation, Kenya

## Abstract

**Background:**

Understanding the availability of rapid diagnostic tests (RDTs) is essential for attaining universal health care and reducing health inequalities. Although routine data helps measure RDT coverage and health access gaps, many healthcare facilities fail to report their monthly diagnostic test data to routine health systems, impacting routine data quality. This study sought to understand whether non-reporting by facilities is due to a lack of diagnostic and/or service provision capacity by triangulating routine and health service assessment survey data in Kenya.

**Methods:**

Routine facility-level data on RDT administration were sourced from the Kenya health information system for the years 2018–2020. Data on diagnostic capacity (RDT availability) and service provision (screening, diagnosis, and treatment) were obtained from a national health facility assessment conducted in 2018. The two sources were linked and compared obtaining information on 10 RDTs from both sources. The study then assessed reporting in the routine system among facilities with (i) diagnostic capacity only, (ii) both confirmed diagnostic capacity and service provision and (iii) without diagnostic capacity. Analyses were conducted nationally, disaggregated by RDT, facility level and ownership.

**Results:**

Twenty-one per cent (2821) of all facilities expected to report routine diagnostic data in Kenya were included in the triangulation. Most (86%) were primary-level facilities under public ownership (70%). Overall, survey response rates on diagnostic capacity were high (> 70%). Malaria and HIV had the highest response rate (> 96%) and the broadest coverage in diagnostic capacity across facilities (> 76%). Reporting among facilities with diagnostic capacity varied by test, with HIV and malaria having the lowest reporting rates, 58% and 52%, respectively, while the rest ranged between 69% and 85%. Among facilities with both service provision and diagnostic capacity, reporting ranged between 52% and 83% across tests. Public and secondary facilities had the highest reporting rates across all tests. A small proportion of health facilities without diagnostic capacity submitted testing reports in 2018, most of which were primary facilities.

**Conclusion:**

Non-reporting in routine health systems is not always due to a lack of capacity. Further analyses are required to inform other drivers of non-reporting to ensure reliable routine health data.

**Supplementary Information:**

The online version contains supplementary material available at 10.1186/s12913-023-09296-9.

## Background

Diagnostic testing is vital to improving the standard of care in any health care system. However, diagnostics account for the biggest disparity in the continuum of care for different health conditions [1, 2]. Access to accurate and timely diagnostics minimises reliance on clinical symptoms alone as the basis for diagnosis. Accurate diagnosis supports more targeted treatments that result in better disease management and effective use of limited resources, particularly in low- and middle-income countries (LMICs) [1, 3, 4]. Therefore, delays in diagnosis caused by limited access or inadequate capacity could undermine efforts made globally to achieve the sustainable development goals (SDGs) on health and lowering disease morbidity and mortality [5, 6].

About half of the world’s population has limited access to diagnostics [1]. However, if this gap is narrowed by 10% across select priority conditions, it is estimated that approximately 1.1 million premature deaths would be averted in LMICs [1]. This emphasises the potential impact of improved access to diagnostics, particularly in primary health care, in accelerating universal health coverage (UHC) [1, 2, 7]. Further, across LMICs, heterogeneities in access to diagnostic tests exist between countries and across facility levels [5, 8]. Although primary healthcare facilities are the first point of care, they have the most significant diagnostic capacity deficit [9]. Only about 19% of the population in LMICs have access to basic diagnostic testing, excluding malaria and HIV. For instance, in 2004–2018, ten LMICs (including Kenya), overall test availability for basic primary care was 19%, while advanced primary care and hospitals were 49% and 68%, respectively [3].

Rapid Diagnostic Tests (RDTs), which are point-of-care diagnostics, provide results with shorter turnaround times than conventional laboratory-based testing and do not require sophisticated equipment [10]. These tests are beneficial for filling gaps in diagnostics in LMICs [6, 9]. Over the years, improvements in diagnostic support have been concentrated on key programmatic areas like HIV, Tuberculosis (TB), and malaria in LMICs [3, 11]. However, this only covers a portion of the broad diagnostic spectrum; thus, substantial availability and quality gaps remain, even for diseases with a high priority for public health [3, 5, 12].To address this problem across countries, since 2018, World Health Organisation (WHO) has published an annual essential diagnostics list (EDL), a suite of recommended diagnostics that should be available at point-of-care, laboratories and across all health tiers to increase timely, evidence-based and life-saving diagnoses [7]. The Lancet Commission further suggests the development of an evidenced-based integrated and tiered network and a national EDL across countries to inform their national diagnostics strategy [1].

A comprehensive understanding of availability of RDTs in LMICs, remains a crucial priority in advancing the UHC agenda [7] and reducing overall accessibility gaps. Routine data [13–15] has been used widely in monitoring progress in health service provision, including laboratory testing across health systems [3]. For example, test volumes of RDTs across health facilities can be compared with the population needing diagnostic testing to determine unmet needs, ensuring efficient use and allocation of resources in the community. However, the utility of routine health data has been impacted by several limitations such as poor reporting and completeness. In such cases, triangulation of facility data with health facility surveys is a valuable data validation approach [3, 16]. In addition, sample health facility surveys offer alternative sources of information in understanding facility capacities beyond facilities’ routine data from the health management information System (HMIS). Such surveys assess the readiness of healthcare facilities to provide services [17], for example, the most recent (2018) Kenya Health Facility Assessment (KHFA), a nationally representative facility assessment that evaluated various aspects of service and diagnostic availability through in-person facility visits [18]. Such surveys provide a rich set of cross-sectional data for a sample of facilities; however, they are conducted infrequently and only focus on select health services.

Routine diagnostic testing data reported monthly by facilities through the HMIS based on the District Health Information Software 2 (DHIS2) platform is more ubiquitously available. Since 2011, the platform has provided routine data on tests done at a facility level every month in Kenya. Despite the availability of routine data across the country for an entire year, data quality has been a concern, primarily due to non-reporting and incomplete entries across health facilities [13, 15, 19–23]. Dealing with these challenges requires an understanding of the causes driving non-reporting. This study triangulated between routine data reported through DHIS2 and a cross-sectional health facility assessment survey to assess whether a lack of diagnostic capacity drove non-reporting in Kenya’s DHIS2 at the facility level for ten RDTs between 2018 and 2020.

## Methods

### Country context

Kenya is in the East Africa region with a total population of 47.6 million, based on its 2019 census, and has an inter-censual growth rate of 2.2% [24]. The average population density was 82 per square kilometre (sq. km) and was highly variable at the sub-national (county) level ranging from less than 20 people per sq. km to over 500 people per sq.km. Population distribution has major consequences on health policy and management decisions relating to allocating resources, distribution of health facilities, and diagnostic availability. Since 2013, Kenya’s health sector has operated under a decentralised governance structure where functions of administration and sub-national health care provision decisions were devolved to 47 county governments. However, the management of tertiary health facilities, regulatory processes, and health policy direction was retained at the national level.

Health provision in Kenya is structured into six tiers of increasingly complex service delivery, namely community-level services (level 1), primary health care consisting of dispensaries, clinics (level 2) and health centres (level 3), secondary and tertiary care comprising sub-county hospitals, medium-sized private hospitals (level 4), primary referral hospitals (level 5), and national referral hospitals (level 6) [25]. Health facilities are owned and managed by the government, non-governmental institutions, faith-based organisations or private-for-profit entities. Medical laboratories in Kenya also fall under similar ownership categories and may be located either within a health facility or as stand-alone facilities, both of which are regulated by the Kenya Medical Laboratory Technicians and Technologists Board (KMLTTB) [13]. The diagnostic tests available in Kenya range from simple RDTs to complex laboratory tests, the availability of which is variable and may correspond to the ownership and level of the health facility.

### Methodology overview

**Fig. 1:**
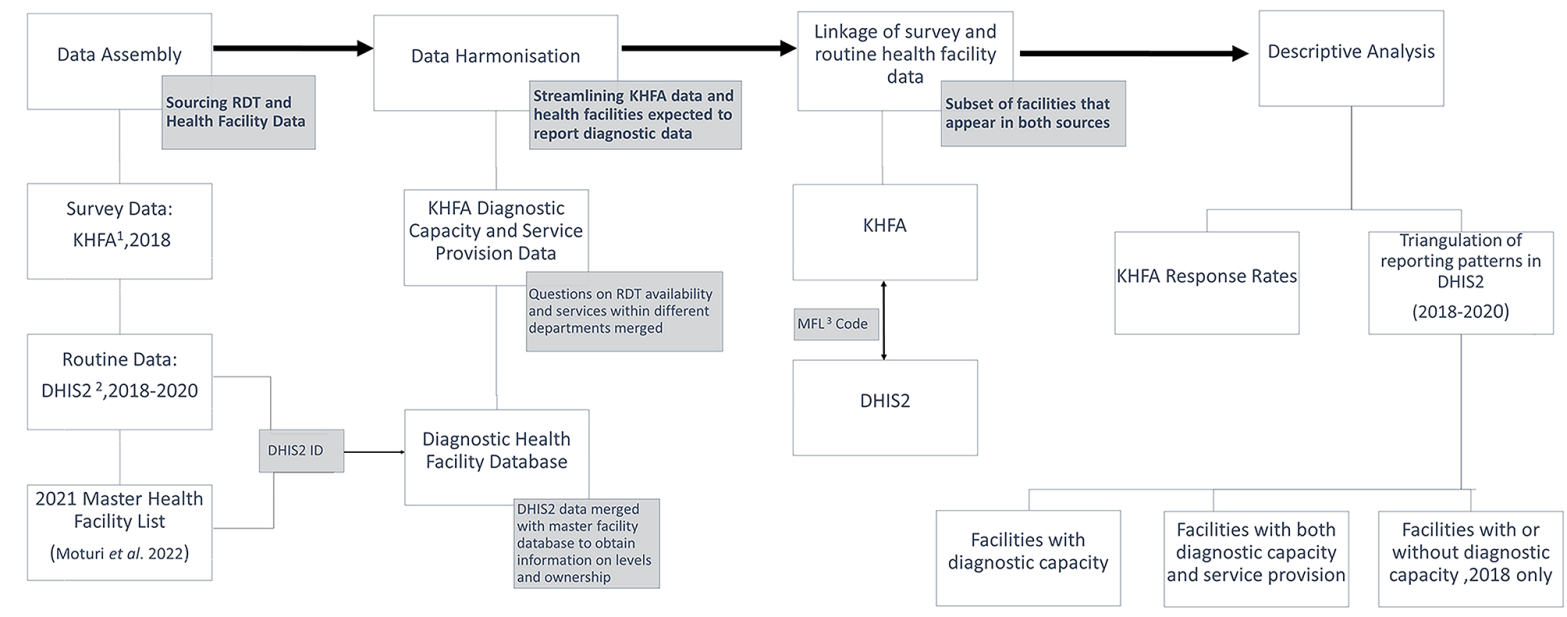
Flowchart outlining overall study approach. (^1^Kenya Harmonised Health Facility Assessment, ^2^District Health Information System version 2, ^3^Master Facility List Code)

This section briefly summarises the study approach in data preparation and analysis with more comprehensive details in the following sections (Fig. 1). The study hypothesis was that all facilities confirmed to provide a service and/or had diagnostic capacity as per the survey, should have submitted a diagnostic report in DHIS2. To test this ,data on ten diseases’ RDTs were assembled at the facility level from DHIS2 for the years 2018–2020 and from the 2018 Kenya health facility assessment survey. The questions within the survey were repeated for different health facility departments and were first harmonised to determine test availability and service provision across the facility. Descriptive analysis was used to summarise the overall diagnostic capacity and service provision at a national level and disaggregated by facility level and ownership for all ten RDTs based on the cross-sectional survey. Following this, a one-to-one comparison was carried out between the KHFA data and the DHIS2 data for each facility. Consequently, the proportion of facilities that submitted a report in DHIS2 was computed for the facilities with confirmed capacity for (i) testing, (ii) or both testing and service provision. Data reported in DHIS2 in 2018 only was also validated against facilities with and without diagnostic capacity as per KHFA.

### Data assembly

#### Routine RDT data

Laboratory testing data for health facilities is submitted to DHIS2 using the Ministry of Health (MoH) reporting tool *MoH 706*, which encompasses 91 diagnostic tests available in Kenya [26]. Guided by the most recent WHO EDL [27] details on assay format for different tests and local clinician knowledge, 18 RDTs were identified from this tool. These RDTs were then compared with tests included in KHFA to obtain a list of RDTs for the triangulation. An RDT was included in the triangulation if reported through DHIS2 and evaluated for diagnostic capacity and/or linked service provision in KHFA.

Based on the KMLTTB checklist, supplemented with local lab technician\technologist knowledge and informed by literature, the facility levels at which each RDT was expected was determined and tests were classified as either common or uncommon [13]. Each RDT was also classified into 4 categories based on the WHO EDL categories namely: clinical chemistry, haematology, sexually transmitted infections and bacteriology, mycology and parasitology.

The triangulation included ten RDTs: malaria, HIV, blood grouping, urine chemistry, Treponema Pallidum Hemagglutination (TPHA), Venereal Disease Research Laboratory (VDRL), Human Chorionic Gonadotropin (HCG-pregnancy), HB estimation, blood sugar and Cryptococcal Antigen (CRAG) tests (Table 1). Eight RDTs that were excluded (oral glucose tolerance test, rheumatoid factor, brucella, H. pylori, hepatitis A, B and C tests and anti streptolysin o titer (ASOT) tests) were reported in DHIS2 but test availability was not assessed in the KHFA. In DHIS2, malaria RDT reports are disaggregated into facility and community health worker (CHW) reports however, this study only utilised facility reports in analysis as test availability specifically for community health workers was not determined in the KHFA.

**Table 1:** Rapid diagnostic tests reported in DHIS2, whose availability was assessed in the 2018 KHFA survey

RDT Test	Health outcome	Common	Level of reporting	WHO EDL classification
HIV	HIV	Y	Level 2–6	Sexually transmitted infections
Venereal disease research laboratory (VDRL)	Syphilis	Y	Level 2–6	Sexually transmitted infections
Blood grouping	Blood Transfusion	Y	Level 2–6	Haematology
HB estimation	Anaemia	Y	Level 2–6	Haematology
Blood Sugar	Diabetes	Y	Level 2–6	Clinical chemistry
HCG (Human chorionic gonadotropin)	Pregnancy	Y	Level 2–6	Clinical chemistry
Urine Chemistry	UTI, Kidney Disease	Y	Level 2–6	Clinical chemistry
Cryptococcal Antigen (CRAG) test*	Cryptococcal Meningitis	Y	Level 2–6	Clinical chemistry
Malaria RDT	Malaria	Y	Level 2–3	Bacteriology, mycology and parasitology
Treponema Pallidum Hemagglutination (TPHA)	Syphilis	N	Level 4–6	Sexually transmitted infections

* Mostly available at facilities offering antiretroviral therapy (secondary facilities), UTI.—Urinary tract infections

Data from DHIS2 were downloaded using MoH authorised login [27] on 25 November 2021. The data for 10 RDTs were extracted monthly at the facility level for 2018–2020. The three-year period was selected to enable accounting for reporting biases in certain months attributable to disruptions such as facility stockouts and the COVID-19 pandemic [15]. It also provided an objective view of the facilities long-term reporting patterns with an endpoint placed in December 2020 to avoid a substantial temporal mismatch with the KHFA.

#### Diagnostic health facility database

To determine health facilities expected to report diagnostic tests, this study utilised data from a recently updated and geocoded database of all public and private health facilities in 2021. The database was assembled using Kenya’s two main lists of health facilities, the Kenya master health facility list (KMHFL) and DHIS2 which were compared, harmonised and merged into one database [28]. Facility data sourced from DHIS2 was merged with this master health facility database, using the unique DHIS2 identifier, to obtain additional details on facility level, type, ownership and Master Facility List (MFL) code which are not available in DHIS2. Exclusions were made for facilities where diagnostic testing is not routinely carried out including medical stores, depots, specialist clinics, treatment centres, and rehabilitation centres. The final database contained a list of all diagnostic health facilities showing the number of monthly RDT tests submitted by facilities and their facility attributes.

#### Kenya harmonised health facility assessment survey

The KHFA conducted from November to December 2018 was a comprehensive survey of health facility capacity across themes of service availability and readiness, quality of care and management. The KHFA used the KMHFL as its sampling frame, comprising 10,535 health facilities. The selection of facilities was stratified across counties, facility type, managing authority and settings (rural or urban), resulting in 2,980 facilities randomly selected. All facilities at levels 5 and 6 and all public level 4 hospitals were included in the sample.

The survey entailed in-person facility visits and interviews with the most knowledgeable staff in each service area. Availability of diagnostic items was determined for the day of the survey and stockouts for the preceding 3 months of the survey period. Test availability and service provision assessment was disaggregated by department thus information was spread out across several questionnaires. Extraction was carried out to obtain each facility’s responses in the following questionnaires with relevant data: laboratory and blood transfusion, emergency, maternal and child health (MCH) and family planning, maternity and newborn, and HIV service area. Data were collected electronically on Android phones for 2,927 (98%) out of the target 2,980 facilities. The specific variables extracted are summarised in Supplementary Tables 1 & 2 .

### Data harmonization

Questions in the KHFA on test and service availability were duplicated across different departments in a facility or disaggregated for select populations (e.g., HIV testing for TB patients, adolescents, children etc.). This phenomenon was further complicated by fragmented data collection during the survey, resulting in variable response rates and occasionally conflicting information in related questions [18]. Consequently, for purposes of triangulation, the study considered the broader availability of a service or test within the facility regardless of department. For this study, the term *diagnostic capacity* is used to refer to RDT availability (both expired and unexpired test kits) within a facility. Further, this study adopts the KHFA definition of *service provision* which referred to a facility’s general capacity to screen for, diagnose and/or treat a given disease [18]. Related questions were thereby referenced and merged to obtain final responses for diagnostic capacity and service provision. Any confirmation of test availability or service provision in pertinent questions was considered definitive (Supplementary Tables 1 & 2 ). After harmonisation, facilities with no response in either category were treated as missing entries and excluded in the consequent analysis.

Utilising the MFL code that uniquely identifies facilities, the harmonised KHFA dataset [18] was linked to the DHIS2 health facility database. A total of 2,821 facilities were matched successfully, excluding 106 facilities, of which 100 health facilities (95 private-for-profit and 5 public health facilities) in KHFA were not registered in DHIS2, and thus data on testing could not be obtained. The other six were specialist facilities ((Ear, Nose and Throat (ENT) and dermatology) and were not expected to conduct diagnostic tests of interest routinely. Private-for-profit health facilities might not be enrolled in DHIS2 since enrolment requires them to submit monthly reports to DHIS2, which might increase workload. On the other hand, public facilities may have experienced delays in registration.

### Triangulation

This study independently analysed the harmonised responses for diagnostic capacity and service provision for each RDT with reports from DHIS2 for 2018–2020 and 2018. Using data collated over three years ensured that controlling for disruptions that might have contributed to non-reporting, such as stockouts or improved reporting in DHIS2 in the post-survey period was possible. Triangulation was carried out in three separate scenarios; a comparison of reporting in DHIS2 (2018–2020) among facilities with (i) diagnostic capacity only and (ii) both confirmed diagnostic capacity and service provision based on KHFA and (iii) a data validation using all responses on diagnostic capacity compared to 2018 DHIS2 reports only. In the second scenario, total RDTs with confirmed diagnostic capacity and service provision data were lower since not all facilities reporting services reported having the corresponding RDTs.

A descriptive analysis of response rates in KHFA for diagnostic availability and service provision questions for each RDT was undertaken as a first step. Thereafter, for each of the two scenarios, among the health facilities with established capacity, the proportion that submitted reports ( > = 1 test) in DHIS2 over the entire 3-year period was computed. Lastly, the study assessed whether facility responses on diagnostic capacity were consistent with reporting patterns in DHIS2 for 2018 alone. All analysis was conducted per RDT at the national level and disaggregated by facility level and ownership using R software environment (Version 4.2) and StataCorp. 2021 [Stata Statistical Software: Release 17. College Station, TX: StataCorp LP].

## Results

**Fig. 2:**
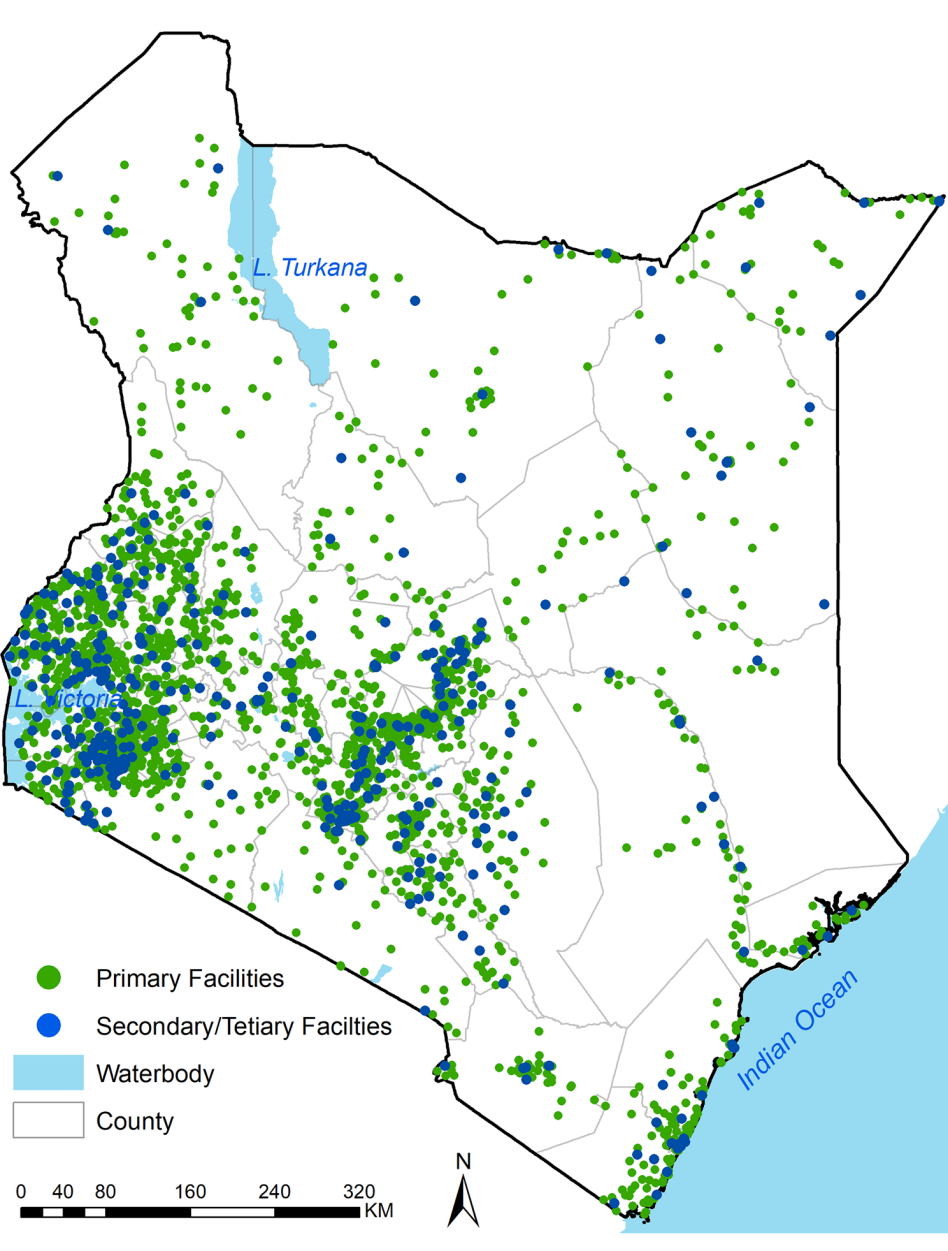
Spatial distribution of 2821 health facilities used in triangulation sampled in the facility survey and registered on DHIS2.

The total number of health facilities used for triangulation was 2,821, geographically distributed across all 47 counties (Fig. 2). This sample represented 21% of all health facilities expected to submit diagnostic reports in the DHIS2 in Kenya. The majority (70%) were public facilities owned by either the government, non-governmental or faith-based organisations, while private-for-profit was 30% (845). The primary health facilities (Level 2–3) were 2,412 (86%), while secondary and tertiary facilities (Level 4–6) were 409 (14%).

The number of health facilities responding to diagnostic capacity and service provision-related questions in the KHFA survey varied by test and by facility (Table 2). Responses related to service provision were available for five RDTs (Table 2). Overall, responses to service provision were above 86% (2,427 facilities) for 6 out of 7 of the services of interest. Responses were highest (100%) for HIV testing for Antenatal Care (ANC), malaria and HIV diagnosis, while the lowest response rate at 71% was for diabetes diagnosis (Table 2). For eight of the ten RDTs, over 79% of facilities reported their diagnostic status. Similar to service provision, malaria and HIV RDTs had the highest response rate of over 96% representing 2,700 out of 2821 facilities forming the sample. The status of CRAG and blood grouping tests were the least reported, with less than 45% of facilities responding (Table 2).

**Table 2:** Response rate to service provision and test availability questions after harmonisation of variables (N = 2821)

RDT	Services available	Services provision (%)	Test availability (%)
Syphilis	Syphilis Diagnosis	86.4	79
Syphilis	Syphilis ANC Testing	86	79
Blood Sugar	Diabetes Diagnosis	71	91
HB Estimation	Anaemia Diagnosis	92	79
Malaria	Malaria Diagnosis	100	97
HIV	HIV Diagnosis	99.8	96
HIV	HIV ANC Testing	100	96
Urine Chemistry	N/A	N/A	91
HCG	N/A	N/A	91
CRAG	N/A	N/A	44
Blood Grouping	N/A	N/A	34

**Fig. 3:**
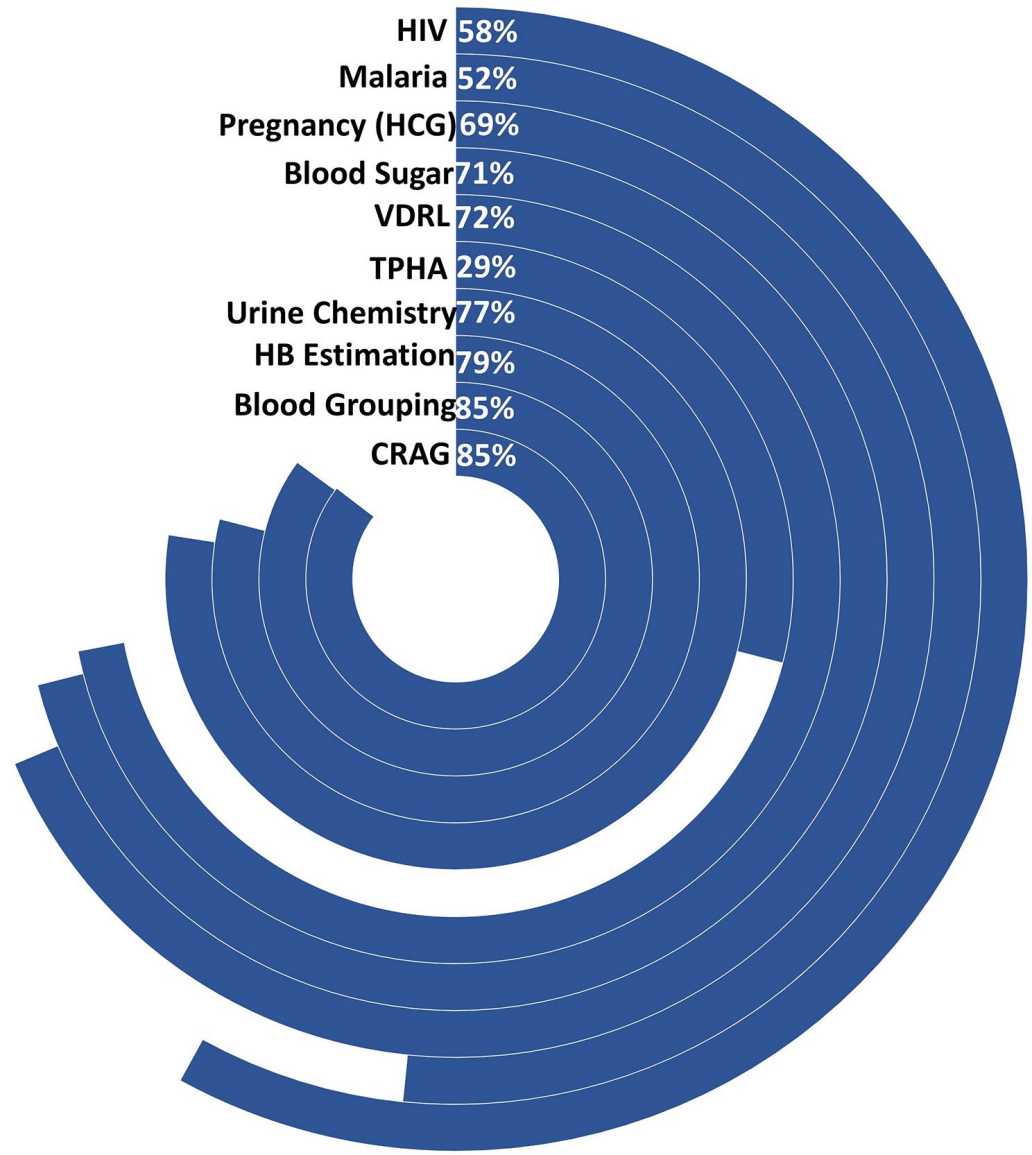
Proportion of health facilities reporting in DHIS2 (blue) among those with confirmed diagnostic capacity from KHFA. The circle size of each test corresponds to the total number of facilities with confirmed capacity, lowest (CRAG) to highest (HIV). Percentage reporting is shown in the figure

Across all common tests, most facilities with confirmed diagnostic capacity as per KHFA submitted reports of testing in DHIS2 (Fig. 3). The proportion of facilities with diagnostic capacity that made a report in DHIS2 varied by test with HIV and malaria having the lowest proportions reporting at 58% and 52%, respectively, while the highest proportions in reporting were for CRAG and blood grouping tests at 85%. The reporting of the other five common tests ranged between 69% and 79%. On the other hand, TPHA (an uncommon test) was not reported in most facilities (71%) despite having reported capacity (Fig. 3).

**Fig. 4:**
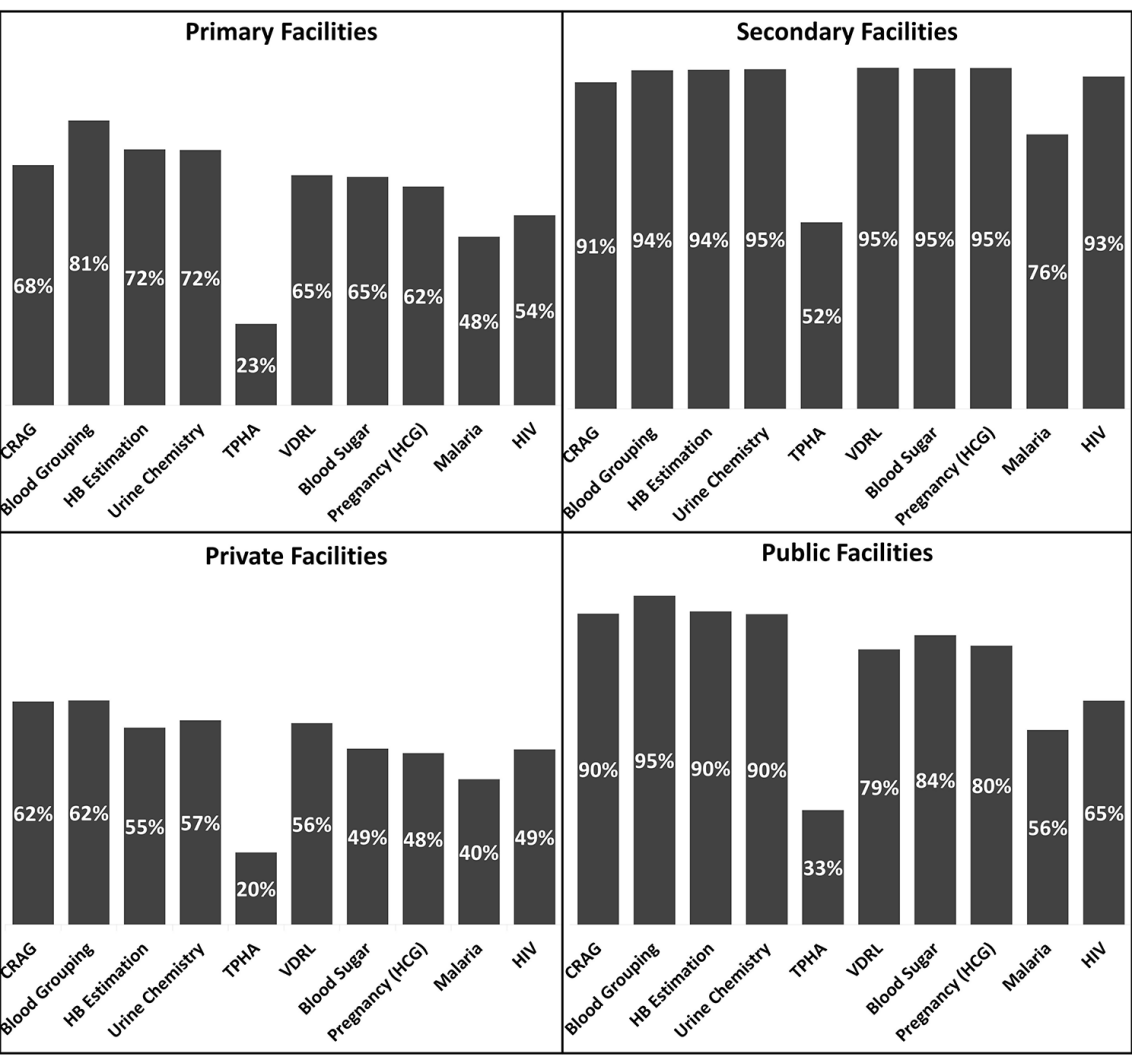
Proportion of health facilities reporting in DHIS2 among those with confirmed diagnostic capacity from cross-sectional survey data. Panels are disaggregated by level, primary (top left) and secondary level (top right) and by ownership, private (bottom left) and public facilities (bottom right)

Heterogeneity was observed in reporting across ownership and facility levels among the facilities with confirmed diagnostic capacity (Fig. 4). The private and primary care health facilities had poorer reporting than public and secondary health facilities. Across all tests, primary facilities with capacity have less than 81% making a report in DHIS2, which is even lower for private facilities with less than 62% reporting (Fig. 4). Comparably, secondary and public facilities each had over 80% of facilities with capacity, reporting to DHIS2 in 6 out of 10 tests. When further disaggregated by ownership, patterns of reporting by primary and secondary facilities reveal similar trends (Supplementary Figs. 3 & 4 ).

Among facilities with diagnostic capacity, reporting in DHIS2 followed the expected trends when analysed by facility ownership status or level. In the case of tests for priority programmes, HIV and malaria, reporting in DHIS2 showed poorer reporting in private (40% and 49%) compared to public facilities (56% and 65%), respectively (Fig. 4, bottom panel). On the other hand, uncommon tests such as TPHA, which had the lowest reporting rates across all facilities (as shown in Fig. 3), and CRAG had significantly better reporting in higher-level facilities (52% and 91%, respectively) where these tests are expected to be available (as illustrated in Fig. 4, top panel) compared to primary level facilities (23% and 68%).

**Fig. 5:**
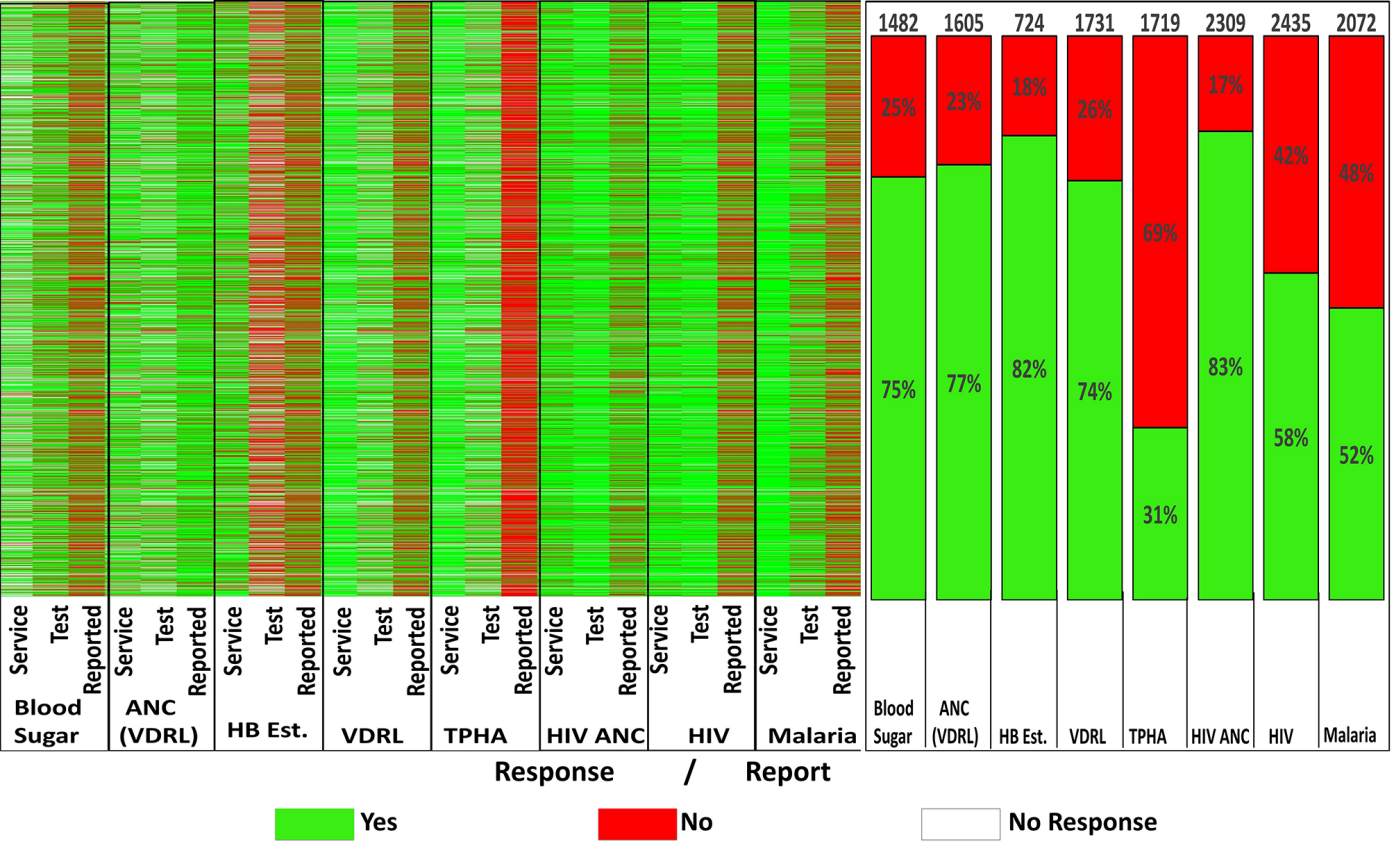
Heat plot of responses to service and test availability questions in the survey (labelled service & test) compared to DHIS2 reports (labelled reported) for each of the 2821 facilities (Panel 1). Panel 2 shows the proportion of health facilities reporting in DHIS2 among those with confirmed service provision and diagnostic capacity from cross-sectional survey data. TPHA is an uncommon Syphilis test

Responses to service provision questions in the survey showed that despite a facility offering a service, it did not always have a corresponding RDT; most visible differences were observed in TPHA (Fig. 5, left panel). Facilities with both the service and the corresponding RDT were highest for HIV, with up to 2,435 (97% of facilities providing HIV services) facilities having both (Fig. 5, left panel). The range of facilities having both service provision and diagnostic capacity across other tests was between 1,482 (89%) for blood sugar and 2,309 (96%) for HIV ANC in 6 tests, with Hb estimation having the least at only 724 (35%). Nationally, among health facilities with both confirmed service provision and diagnostic capacity as per the survey, the proportions of facilities submitting test reports to DHIS2 ranged from 52 to 83% across common tests. Notably, HIV and Malaria RDTs had substantially low reporting rates in DHIS2 (58% and 52%) despite recording high diagnostic capacity and the largest sample size in the KHFA (93% and 76%), respectively. Similar to previous trends, the lowest reporting was associated with TPHA.

**Fig. 6:**
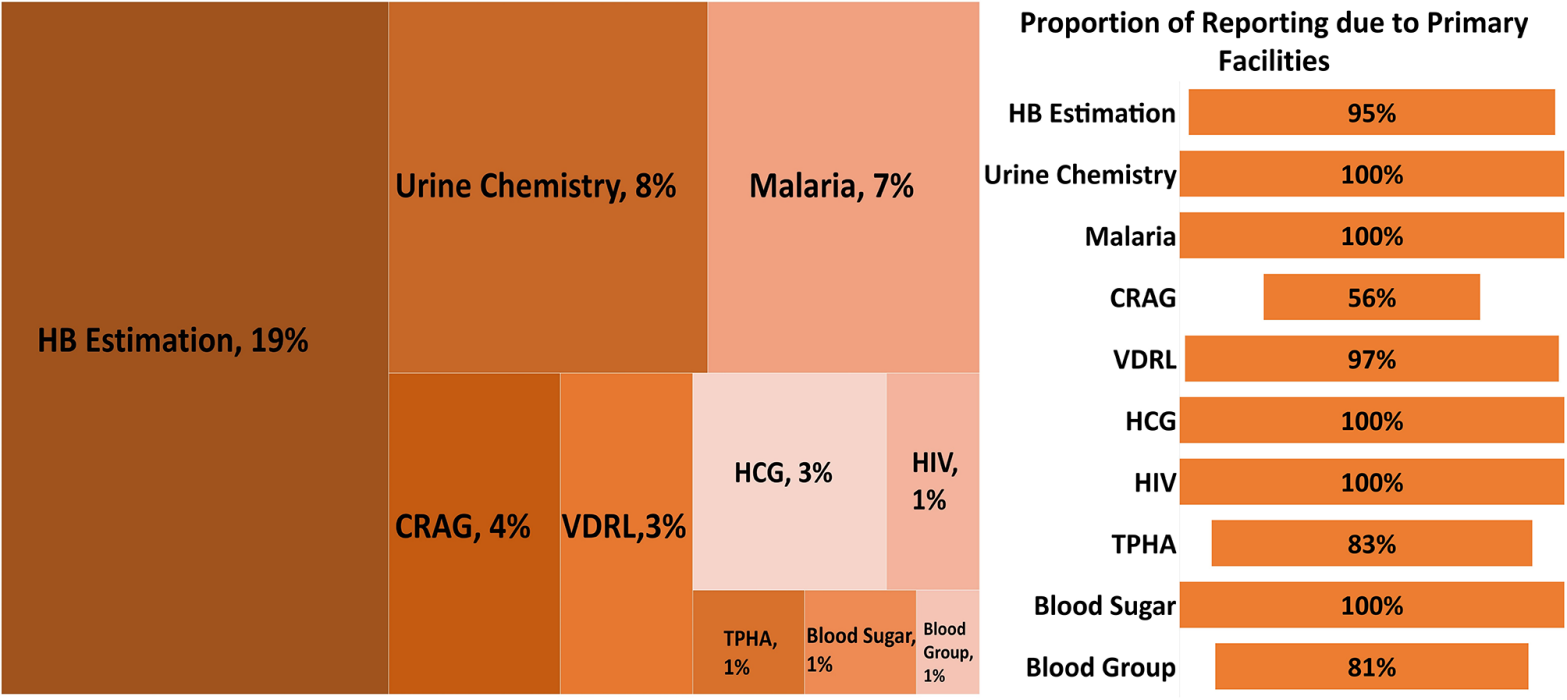
Proportion of health facilities reporting tests performed in DHIS2 despite confirmed lack of diagnostic capacity as per a cross-sectional survey for 2018 only, N = 2821(Panel 1). Panel 2 shows the proportion of reporting in Panel 1 that is due to primary facilities

When data on diagnostic capacity from the survey were compared to reports in DHIS2 for 2018 only, there were minor differences in the information provided by the two sources. Albeit a small proportion, several health facilities without the diagnostic capacity for specific tests, according to the survey, reported conducting the test in DHIS2 in 2018. This varied across RDTs, with the most significant inconsistency exhibited in HB estimation, with 19% of facilities without recorded testing capacity in the survey reporting in DHIS2 (Fig. 6).

## Discussion

Routine health data is essential for quantifying health care utilisation, estimating the reach of interventions in the community and monitoring progress toward national and global targets such as SDGs [29]. However, data quality concerns, primarily due to the non-reporting of health facilities, have continued to persist, impacting the accurate assessment of the performance of a country’s health system [15, 19–21, 23]. These concerns become even more significant when estimating the current supply and demand for point-of-care testing to guarantee an adequate diagnostics supply. Consequently, to address quality concerns specifically data completeness in routine data reported through DHIS2, this study sought to establish whether the non-reporting of health facilities in DHIS2 is due to a lack of capacity triangulating diagnostic test reports made in DHIS2 with health capacity information from a comprehensive facility survey, KHFA.

Our findings show that most facilities that offer both diagnostic services and have requisite RDTs reported testing on the DHIS2 platform. However, about 17–48% of the facilities did not report, and yet they could conduct the common tests (Fig. 5). More importantly, despite routine tests, HIV and malaria RDTs had 42–48% of facilities with RDT kits available at the time of the survey failed to report testing in DHIS2 over three years. When the analysis was restricted to data from 2018 to ensure congruence in temporal comparison, a similar pattern was observed, with 38% and 43% of facilities failing to report for HIV and malaria, respectively. Applying the same temporal restriction (2018), across the other RDTs, facilities failing to report despite having RDT kits were heterogeneous, ranging from 2% (CRAG) to 55% (TPHA). Further, reporting is driven by level, and facility ownership as primary health facilities and private-for-profit owned facilities account for the largest share of poor reporting despite having capacity consistent with findings in other studies [30, 31].

Although survey data is constrained since it only provides a cross-sectional picture, its high degree of accuracy can be used to understand better non-reporting problems related to the RDTs in DHIS2 through triangulation. The survey data have shown that RDT kits are routinely available within health facilities but may not necessarily be captured in the routine health information system reporting. Therefore, relying on routine data only to inform the availability of tests may underestimate the actual diagnostic capacity among health facilities, emphasising the need for validation and triangulation with other data sources. Our results are consistent with a study in which no health facility reported diagnostic testing for malaria in DHIS2, despite 90% of health facilities having the diagnostic capacity and 40% confirming malaria-positive cases [32].

The historical investment [3, 33] in high-priority diseases such as HIV and malaria was apparent as most facilities (> 76%) reported having these RDT kits (Supplementary Fig. 1 ). For such priority programmes, routine data presents an invaluable surveillance source, facilitating accurate assessment of current disease prevalence to determine intervention coverage. Therefore, it would be expected that the reporting pattern in DHIS2, particularly for these two tests, would correspondingly be as high as the testing capacity; however, a high non-reporting rate was observed. Multiple programmes are keen to track HIV and malaria in Kenya and may have introduced parallel reporting channels within DHIS2. Thus, the lack of integration of monthly reporting tools and the elimination of old reporting tools after updates to remove redundancies within DHIS2 could contribute to poor reporting within the *MoH 706 tool* [34]. Further, technical efforts such as Electronic Medical Record Systems (EMRs) can potentially improve facilities’ reporting performance [35, 36]. However, the lack of interoperability between EMRs and DHIS2 to allow seamless data transmission remains challenging [30]. Additionally, testing and reporting within EMR and DHIS2 are subject to human behaviour [37, 38]. The reliance on syndromic diagnosis may result in the under-utilisation of diagnostic tests even where they are already available [39]. Therefore, there is a need to address challenges such as parallel reporting channels and interoperability between systems to obtain reliable diagnostic metrics in pursuing robust metrics to track the SDGs and the UHC agenda.

Compared to the secondary health facilities, primary facilities had poor reporting in the DHIS2 for facilities with capacity. This might be ascribed to the fact that not all primary facilities have access to the DHIS2 system; instead, some rely on the resources available at the sub-county level. Further, the facility records data on manual forms that are later aggregated to the platform, which delays validation checks to ensure data quality [30]. The manual entry may have contributed to the instances where facilities with no testing kits submitted a report of testing in DHIS2, which was also largely prevalent in primary health facilities (Fig. 6, Panel 2). In some cases, the sub-county health records officer (HRIO) tasked with data entry may be overwhelmed by the number of facilities reporting contributing to delays and errors [30, 40, 41]. This and other potential drivers may potentially account for some portion of the primary facilities submitting reports to DHIS2, yet they had no diagnostic capacity (Fig. 6, Panel 2) [30, 40, 41]. The possibility of erroneous reports driven by data entry errors or pressure to meet performance targets or the quality of KHFA requires further examination.

Among the health facilities, private facilities had poorer reporting rates relative to the public facilities even when further disaggregated by levels (Supplementary Fig. 3 , Panel 1). This is directly linked to the diagnostic capacity that is skewed towards public facilities. For all RDTs, 63-84% of facilities in the public sector had diagnostic capability compared to only 16–37% in the private sector (Supplementary Fig. 2 ). Private health facilities might be hesitant to adopt DHIS2 if the benefits gained do not supersede their existing systems contributing to poor reporting in DHIS2 [38]. Ensuring access to essential RDTs cannot be met solely through the public sector. However, the inequality in capacity between public and private facilities evident from the survey data highlights an existing capacity gap that needs to be filled to increase the availability of RDTs in the community. Kenya’s Vision 2030 set forth the agenda to expand public-private partnerships in healthcare service provision to ensure adequate health services for the growing population [42]. In addition, a better understanding of the sub-national level diagnostic landscape will better tailor and guide public-private partnerships where needed.

DHIS2 reporting for RDTs, particularly malaria and HIV, is likely to have been impacted by stockouts in 2018. These two RDTs had the highest stockout rates, 31% and 13%, respectively, out of the six RDTs where the stock was evaluated (Supplementary Table 3 ). Over half of the facilities with stockout (51% for malaria and HIV) in 2018 did not submit reports to DHIS2. Dealing with systemic stockout issues, such as supply chains, through simple approaches like SMS reminders [43] might reduce the problem. Resultantly, RDTs will be available when needed by patients and may increase the likelihood of reporting utilization.

To optimise the role of RDTs in supporting clinical decisions and filling the diagnostic gap in LMICS, an assessment of the availability of tests is critical. Kenya has a substantial availability of RDTs (hence diagnostic capacity) across the health system. However, the availability varied between RDT type and facility. Across all the RDTs, diagnostic capacity was high (at least twice) in primary facilities (over 67%) when compared to the secondary facilities (less than 33% except CRAG). These results show that primary public facilities could be the largest consumer of RDTs attributable to better health accessibility (affordable, available, spatially accessible) as most citizens’ first point of contact.

Where the health sector faces enormous human resource gaps compounded by funding and infrastructure limitations (8), RDTs are a sustainable solution. However, comparing RDT volumes consumed across the health system versus those needing diagnostic testing is necessary to determine if these RDTs satisfy the community’s needs. Consequently, in the context of limited resources in LMICs, such an analysis would align disease burden, healthcare utilisation, and inequities in diagnostic access for targeted resources for RDTs. Therefore, focusing investment on RDTs may offer the most significant opportunity to bridge the health system diagnostic gap as they are easily implemented in rural and primary health care contexts while requiring minimal technical training to perform and interpret the results [8, 10]. Support by government and donors in training and through entrenching the use of data in decision-making would further incentivise reporting and strengthen routine health systems.

In Kenya, several other variables besides a lack of capacity for health facilities have contributed to non-reporting. First, zero reports (no testing) in DHIS2 are usually converted to missing values by the DHIS2 system [13, 19]. This makes it impossible to distinguish between zero reports and non-reporting. An assumption is thus made that any missing value in the dataset was not reported for that period which inflates non-reporting rates. Second, it is also possible that non-reporting in DHIS2 for some tests may have been triggered by clinicians’ preference for alternative tests despite KHFA demonstrating confirmed service availability. For instance, a full hemogram is a frequently utilized test panel that provides a wide range of haematological parameters, including Hb levels that haemoglubinometers would have rapidly offered.

Further, with the introduction of Kenya’s devolved health structure [40], an eventual increase in the number of sub-counties (from 185 to 316) may have resulted in constrained Health Information System (HIS) resources [44, 45]. Moreover, poor induction of health officers during the transition period, particularly on the new system and reporting, could cause some of the data quality issues currently experienced with the DHIS2 system [45, 46]. In addition, poor logistical infrastructure, including inadequate computer access, internet connectivity, and electricity services, remains a challenge, especially in lower-level health facilities [8, 15, 30, 47]. For instance, lagging internet connection and system downtimes have led to delays in reporting and submission of reports [30, 46]. The lack of electricity has also hindered health facilities’ direct implementation of data entry [47]. Other elements contributing to reduced reporting and requiring further investigation include health workers’ strikes [22, 48], low motivation for health workers, and limited human resources.

There is potential to leverage DHIS2 data to improve data reporting, completeness, and timeliness [30, 47, 49]. The DHIS2 platform has inbuilt data quality checks and customizable data analysis features that encourage data use at the point of collection and the lowest level [47] However, the culture of using DHIS2 information for decision-making still needs improvement [47, 50]. For example, a study conducted in Nairobi shows that health facilities were not utilizing performance reports on completeness and timeliness to identify those performing poorly [30, 50]. In addition, the people involved in data collection and entry differ from those involved in data analysis and the generation of reports [50]. This has influenced the perception that the use of data for decision-making is centralized at the national and sub-national levels and that health facilities are limited to data input. Further, a lack of technical skills and training to effectively use DHIS2 may limit health workers’ ability to analyze and interpret results and worsen by restricting access rights to the DHIS2 system to higher-level facilities and sub-county officials [30]. Efforts need to be made to enhance collaboration across all cadres of health information systems to facilitate good feedback mechanisms for those collecting and transmitting DHIS2 data [30, 50]. Training on data literacy would also enhance health workers’ sense of data ownership over the reporting and use of DHIS2 data [47, 50, 51].

## Limitations

In DHIS2, it is impossible to distinguish between zero and missing reports as both are recorded as blanks that may inflate non-reporting. Therefore, in this work, it was that assumed a test report was submitted if at least 1 test was conducted. Triangulation included DHIS2 data for 2020, which may have lower reporting due to disruption during the COVID pandemic; however, the 2018/2019 patterns did not differ from those of 2020. Our sample size for triangulation excluded 106 facilities from the cross-sectional survey as they were not registered on DHIS2. Hence the platform does not capture all functional facilities that are expected to report data. Delay in reporting where reports were submitted after the data was downloaded from DHIS2 may have affected the analysis. Survey questions on service availability for malaria and diabetes were framed as a combination of offering diagnosis and/or treatment. It was assumed an affirmative response included diagnosis based on diabetes management requiring frequent blood sugar checks and wide availability of malaria RDTs due to its priority status. Several RDTs had a small sample of facilities that responded during data collection in KHFA; for example, blood grouping had only 34% of facilities responding. Therefore, the triangulation was informed by an inadequate sample size for several RDTs.

## Conclusion

Data gaps in routine systems have significant implications in informing diagnostics resource allocation. To understand the causes of such gaps, alternative sources such as surveys offer a validation measure but are also imperfect given variable response rates. This study provides evidence that non-reporting in routine health systems is not always due to a lack of capacity and thus further interrogations and analysis are required to inform other drivers of non-reporting and appropriate actions to ensure high data quality and reliable routine health systems.

## Electronic supplementary material

Below is the link to the electronic supplementary material.

Supplementary Material 1

## Data Availability

The datasets supporting the conclusions of this article are available online with authorized access provided by the Kenya Ministry of Health; DHIS2 portal (https://hiskenya.org/dhis-web-commons/security/login.action) and the national master health facility portal (http://kmhfl.health.go.ke/#/home). The Ministry of Health provided the KHFA dataset. The datasets used and/or analyzed during the current study are available to others from these sources through the Ministry of Health Kenya.

## List of abbreviations

ANC: Antenatal Care
ASOT: Anti Streptolysin O Titer
CHW: Community Health Worker
CRAG: Cryptococcal Antigen
DHIS2: District Health Information Software version 2
EDL: Essential Diagnostics List
EMR: Electronic Medical Record
ENT: Ear Nose and Throat
HCG: Human Chorionic Gonadotropin
HRIO: Health Records Information Officer
HMIS: Health Management Information System
HIV: Human Immunodeficiency Virus
KHFA: Kenya Harmonised Health Facility Assessment
KMHFL: Kenya Master Health Facility List
KMLTTB: Kenya Medical Laboratory Technicians and Technologists Board
LMIC: Low- and Middle-Income Country
MCH: Maternal and Child Health
MFL: Master Facility List
MOH: Ministry of Health
RDT: Rapid Diagnostic Test
TB: Tuberculosis
UHC: Universal Health Coverage
SDG: Sustainable Development Goals
TPHA: Treponema Pallidum Hemagglutination
UTI: Urinary Tract Infection
VDRL: Venereal Disease Research Laboratory
WHO: World Health Organisation
