# Supplementary material for: Investigating rapid diagnostic testing in Kenya’s health system, 2018–2020: validating non-reporting in routine data using a health facility service assessment survey
Source: BMC Health Serv Res. 2023 Mar 30;23:306. doi: 10.1186/s12913-023-09296-9 (PMC10061357; doi:10.1186/s12913-023-09296-9)
Supplement: Supplementary file 1 — Supplementary Material 1 [file 12913_2023_9296_MOESM1_ESM.docx]

**Reporting of rapid diagnostic testing in Kenya’s health system supplementary file**

This supplementary file contains 4 figures and 3 tables detailing the harmonisation process used in KHFA survey, stockout responses and additional figures of reporting patterns and diagnostic capacity at different disaggregations.

**Supplementary Table 1: Harmonization of duplicated service provision variables in KHFA to obtain final service provision status**

| **RDT** | **Questions on Service Provision** | **Harmonised Variable** |
| --- | --- | --- |
| **HIV** | Availability of services offered to victims of partner violence - Rapid HIV test | Does facility offer HIV diagnosis |
|  | Do providers screen or test TB patients for HIV |  |
|  | Does facility offer HIV testing services |  |
|  | Does this facility provide HIV testing services for older children 5-9 years |  |
|  | Does facility provide HIV testing services for minor adolescents |  |
|  | Does facility ever provide HIV testing services for children under 5 years |  |
| **HIV ANC** | Availability of PMTCT services - Provide HIV testing services to all pregnant wo | Does facility offer HIV diagnosis in ANC |
|  | Availability of additional PMTCT services - Offer repeat testing for HIV negative |  |
|  | Availability of routine PMTCT services during delivery - HIV test provided |  |
|  | Availability of ANC services - HIV test |  |
|  | Does facility offer HIV testing or other services to ANC clients |  |
| **Malaria** | Service available - Diagnose and treat malaria in children | Does facility offer malaria diagnosis |
|  | Does facility offer diagnosis and/or treatment of malaria |  |
|  | Do providers in facility diagnose malaria |  |
| **CHEW Malaria** | Does facility have links with CHWs for any malaria-related services | Does facility have links with CHWs |
| **Syphilis** | Does facility offer diagnosis and/or treatment of Syphilis | Does facility offer syphilis diagnosis |
| **Syphilis ANC** | Availability of ANC services - Syphilis test (rapid or VDRL) | Does facility offer syphilis diagnosis in ANC |
| **HB Estimation** | Service available- diagnose and treat anaemia | Does facility offer anaemia diagnosis |
| **Blood Sugar** | Do providers diagnose and/or manage diabetes | Does facility offer diabetes diagnosis |

**Supplementary Table 2: Harmonisation of duplicated test availability variables in KHFA to obtain final diagnostic capacity status**

| **RDT** | **Questions on Test Availability** | **Harmonised Variable** |
| --- | --- | --- |
| **Malaria** | Availability of diagnostic tests available in emergency area - Malaria RDT | Malaria Test Availability |
|  | Availability of malaria rapid diagnostic test |  |
|  | Does facility have malaria rapid diagnostic tests (RDTs) kits w/valid exp. date |  |
| **Syphilis** | Syphilis test observed - VDRL test kit | VDRL Test Availability |
|  | Availability of Rapid Syphilis Test |  |
|  | Availability of Rapid Syphilis Test | TPHA Test Availability |
|  | Syphilis test observed - Treponemal specific tests (FTA-Abs) |  |
| **Blood Sugar** | Availability of diagnostic tests available in emergency area - Glucose | Blood Sugar Test Availability |
|  | Availability of rapid test Blood Sugar |  |
|  | Rapid test for glucose |  |
| **HB Estimation** | Handheld test for anaemia | HB Estimation Test Availability |
|  | Availability anaemia test -HemoCue |  |
| **HIV** | Availability of diagnostic tests available in emergency area - Rapid HIV testing | HIV Test Availability |
|  | Availability of rapid test HIV |  |
|  | Does facility have HIV rapid test kits in stock |  |
| **Urine Chemistry** | Availability of rapid test Urine Dipstick 3 | Urine Chemistry Test Availability |
|  | Availability of rapid test Urine Dipstick 9 |  |
|  | Availability of diagnostic tests available in emergency area - Urine dipstick |  |
| **HCG** | Availability of diagnostic tests available in emergency area - Urine pregnancy | Pregnancy Test Availability |
|  | Availability of rapid test Pregnancy |  |
| **CRAG** | Availability of CRAG Test Assay Kit | CRAG Test Availability |
|  | CRAG test availability |  |
| **Blood Grouping** | Cardiac marker test availability - ABO blood grouping testing | Blood Grouping Test Availability |

**Supplementary Table 3: Health facilities per RDT that responded to stock-out**

| ID | Rapid diagnostic Test (RDT) | Responded to test availability | Responded to test availability & had a stock out (%) | Stock out with no DHSI2 report (%) |
| --- | --- | --- | --- | --- |
| 1 | Malaria | 2738 | 839 (31%) | 429 (51%) |
| 2 | HB estimation | 2232 | 96 (4%) | 18 (19%) |
| 3 | Blood sugar | 2581 | 72 (3%) | 18 (25%) |
| 4 | HIV | 2714 | 341 (13%) | 173 (51%) |
| 5 | Urine chemistry | 2581 | 116 (4%) | 27 (23%) |
| 6 | HCG | 2581 | 141 (5%) | 47 (33%) |

Proportion of those with stock-outs (column 4) and facilities that failed to submit a report to DHIS2 among those with stockouts (column 5) in 2018

**Supplementary Figure 1: Diagnostic capacity as per cross- sectional survey among facilities responding to questions on RDT availability**

**
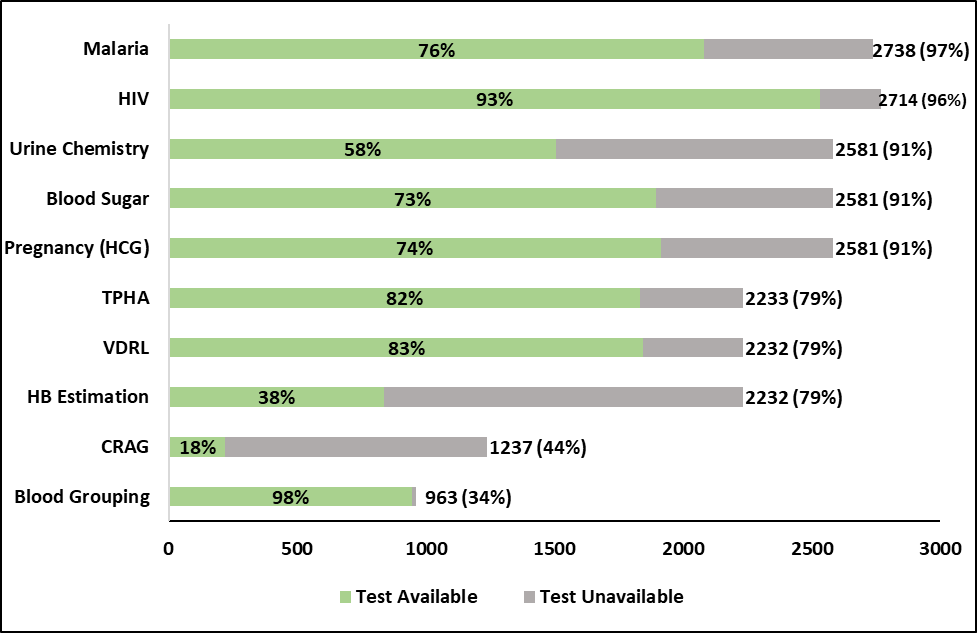
**

**Supplementary Figure 2: Diagnostic capacity as per cross- sectional survey among facilities responding to RDT availability questions ownership.**

**
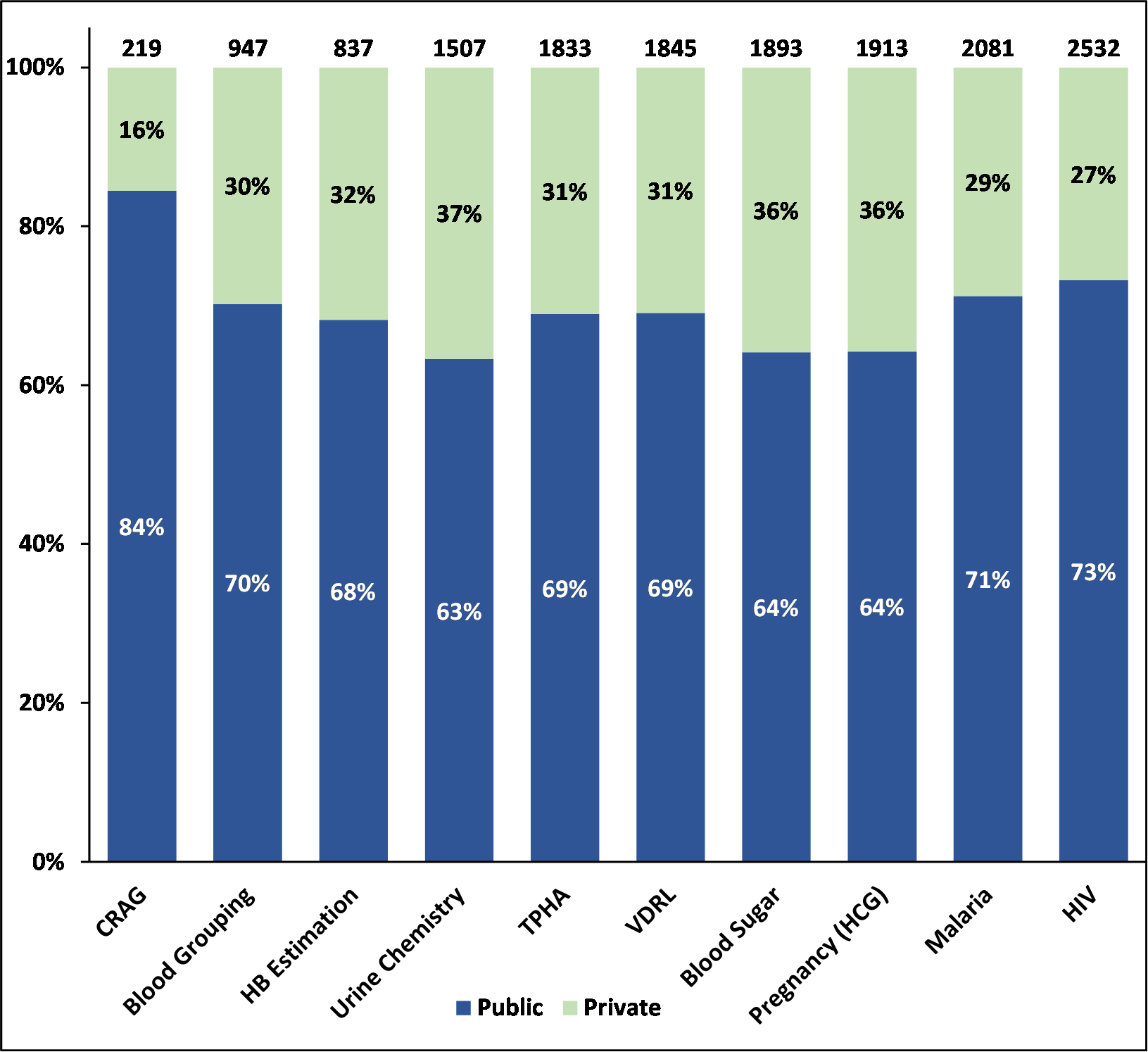
**

**Supplementary Figure 3: Proportion of primary facilities with capacity that submit a report to DHIS2**


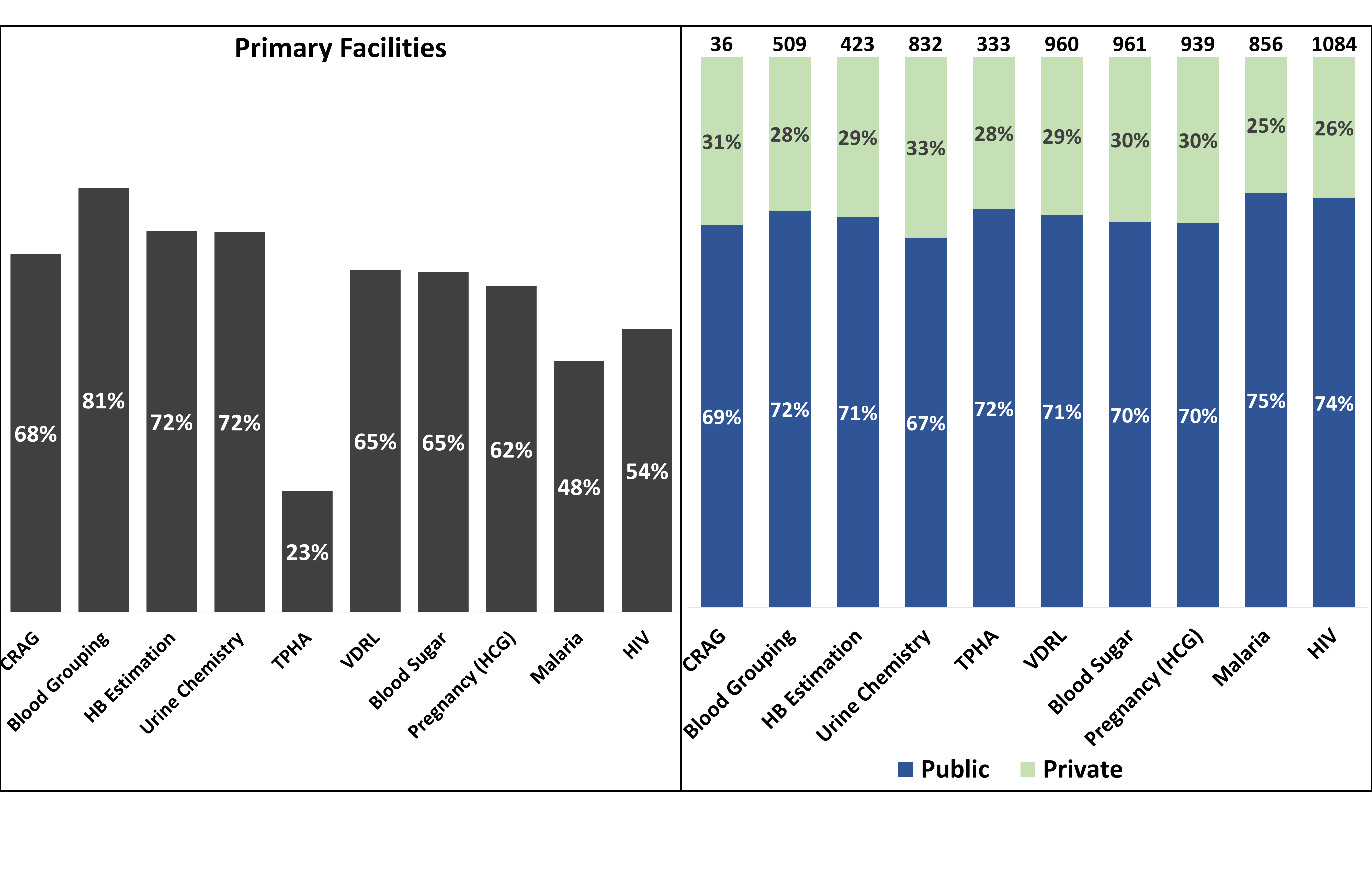


Panel 1 represents primary facilities that submit reports overall. Panel 2 disaggregates reporting among primary facilities by ownership

**Supplementary Figure 4: Proportion of** **secondary/tertiary facilities with capacity that submit a report to DHIS2**


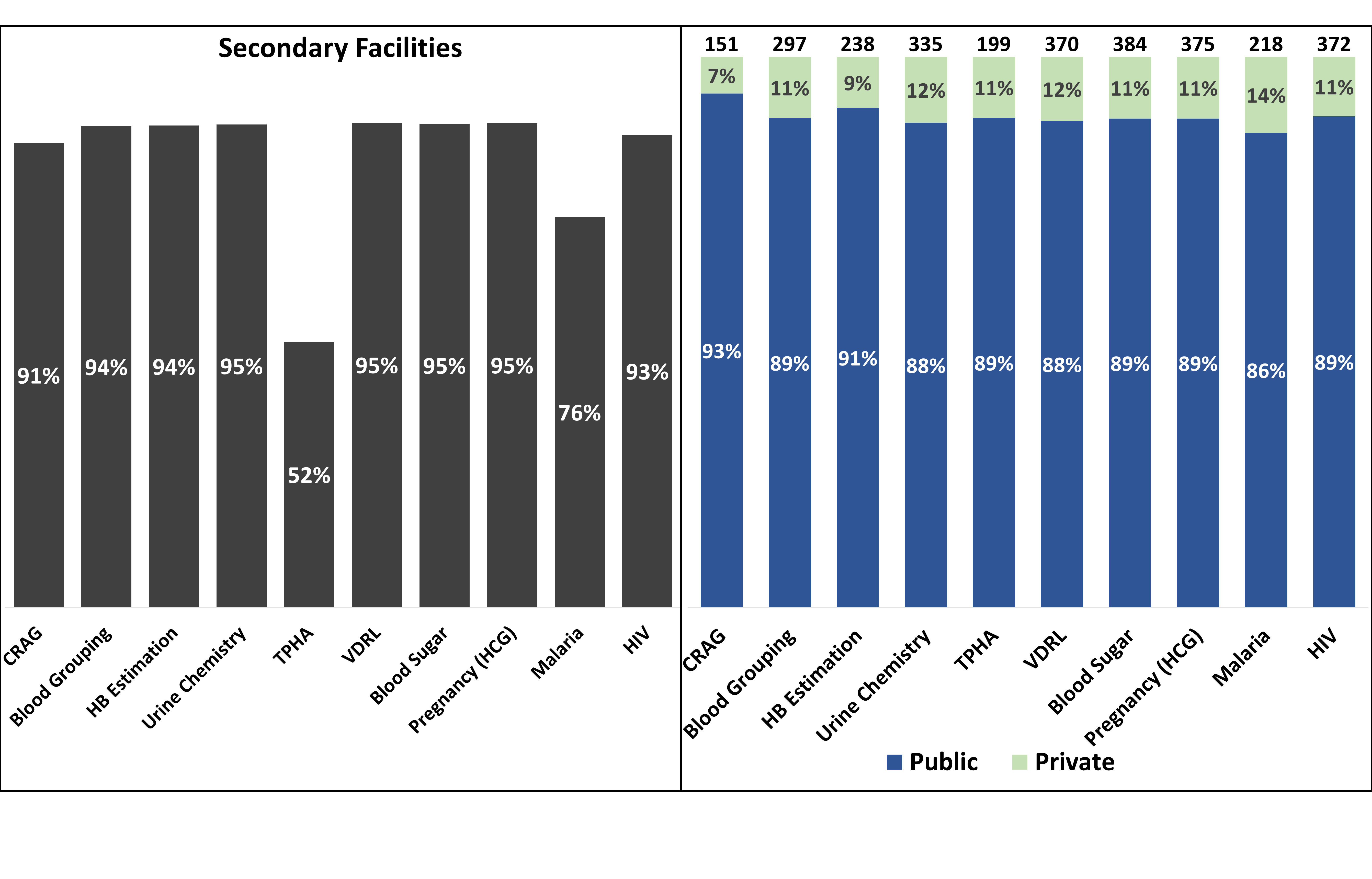


Panel 1 represents secondary/tertiary facilities that submit reports overall. Panel 2 disaggregates reporting among secondary/tertiary facilities by ownership
